# Design and Fabrication of a New Wearable Pressure Sensor for Blood Pressure Monitoring

**DOI:** 10.3390/s21062075

**Published:** 2021-03-16

**Authors:** Marian Ion, Silviu Dinulescu, Bogdan Firtat, Mihaela Savin, Octavian N. Ionescu, Carmen Moldovan

**Affiliations:** National Institute for Research and Development in Microtechnologies, 126A, Erou Iancu Nicolae Street, 077190 Bucharest, Romania; silviu.dinulescu@imt.ro (S.D.); bogdan.firtat@imt.ro (B.F.); mihaela.savin@imt.ro (M.S.); octavian.ionescu@imt.ro (O.N.I.); carmen.moldovan@imt.ro (C.M.)

**Keywords:** pressure sensor, flexible, microfluidic, wearable, healthcare

## Abstract

In recent years, research into the field of materials for flexible sensors and fabrication techniques directed towards wearable devices has helped to raise awareness of the need for new sensors with healthcare applicability. Our goal was to create a wearable flexible pressure sensor that could be integrated into a clinically approved blood pressure monitoring device. The sensor is built from a microfluidic channel encapsulated between two polymer layers, one layer being covered by metal transducers and the other being a flexible membrane containing the microfluidic channel, which also acts as a sealant for the structure. The applied external pressure deforms the channel, causing changes in resistance to the microfluidic layer. Electrical characterization has been performed in 5 different configurations, using alternating current (AC) and (DC) direct current measurements. The AC measurements for the fabricated pressure sensor resulted in impedance values at tens of hundreds of kOhm. Our sensor proved to have a high sensitivity for pressure values between 0 and 150 mm Hg, being subjected to repeatable external forces. The novelty presented in our work consists in the unique technological flow for the fabrication of the flexible wearable pressure sensor. The proposed miniaturized pressure sensor will ensure flexibility, low production cost and ease of use. It is made of very sensitive microfluidic elements and biocompatible materials and can be integrated into a wearable cuffless device for continuous blood pressure monitoring.

## 1. Introduction

Blood pressure (BP) is one of the vital parameters that is checked to identify any global issue at the patient level. Techniques and tools to achieve this monitoring have not changed dramatically since the use of sphygmomanometers by practitioners. This technique relies on the use of a stethoscope and the listening for Korotkoff sounds to determine systolic and diastolic pressure values [1,2,3]. This gold-standard technique is based on the oscillatory method first described by Marey in 1876 [4].

Once the mercury-based gold standard was banned (the auscultatory method of using a mercury sphygmomanometer) [5], some other sphygmomanometers [6] combining oscillatory characteristics together with electronic compliance with the help of algorithms, was developed.

Oscillatory methods are very well suited to monitoring blood pressure at the brachial artery when patients are calm [7,8]. Sphygmomanometer cuffs can easily be attached and removed by the patient. However, the oscillations used to determine the systolic and diastolic pressures are highly affected by low frequency mechanical vibrations, especially those produced by movements during daily physical activity [9]. The algorithms used to interpret the oscillations and Korotkoff sounds vary from device to device, which has settled the need for a new gold standard for these techniques [10,11].

Alternative techniques use ultrasound and the Doppler effect [12]. As the cuff is deflated, the walls of the artery move at systolic pressure, causing a phase change for the reflected ultrasound. The contraction of arterial motion reflects the diastolic pressure [13].

A later interesting technique is the Penaz finger-cuff method [14,15] which detects arterial pulsation in a finger using a photo-plethysmograph (PPG) under a pressure cuff. A photoplethysmogram (PPG) is based on pulse oximetry by detecting changes in the volume of peripheral blood circulation [16]. Being in continuous development, wristwatch devices for blood pressure monitoring are considered to be very comfortable for daily use. They were developed based on an oscillometric method using a cuff limited to the circumference of the wrist. (Omron Healthcare) [17]. Devices of this type require only an interface between the PPG sensor and the wrist surface, but instead of measuring blood flow in the radial artery, they provide information about changes in the volume of capillaries on the wrist surface [18]. To improve the accuracy of measurements made by such devices, many tests have been done in recent years on the integration of machine learning and deep neural networks [19]. On the same note, the standard of the Institute of Electrical and Electronics Engineers for cuffless blood pressure measurement devices (IEEE Std 1708) specifies the need for a calibration process at the validation stage precisely to estimate and recognize unknown parameters [20]. A device that measures and monitors blood pressure and has a finger cuff and an infrared plethysmograph has recently appeared on the market (Finapres, Finapres Medical Systems BV) [21,22]. Although it has a smaller cuff, the device is sensitive to movement and cannot provide continuous data for daily use, so it cannot feasibly be used for long-term BP measurement. Therefore, our goal was to develop a flexible pressure sensor that can be integrated into a wearable medical device for continuous monitoring and detection of BP problems such as hypertension. The sensor aims to have the following characteristics: flexibility, low manufacturing costs, ease of use, highly sensitive microfluidic elements, and biocompatible materials. It is well known that material properties determine a device’s performance, such as involuntary folding and bending without affecting other performances that depend on the characteristics of the materials. Materials for flexible devices should not only have adequate elasticity but also high mechanical and electrical performance. There are different flexible substrates used for sensors applied in different fields. Among these we mention polyethylene terephthalate (PET) film, which is widely used due to its low price and properties like transparency, hardness, good mechanical strength and temperature resistance [23,24]. Polydimethylsiloxane (PDMS) is considered to be the best choice for elastic substrates because of its excellent flexibility and high thermal and dielectric properties [25]. For transducers, metallic (e.g., Au, Ag, Al and Cu) nanoparticles are considered basic materials for flexible sensors due to their remarkable electrical conductivity [26].

The main advantages of our novel wearable pressure sensor are its use of flexible, biocompatible materials with low manufacturing costs, and the use of a low-viscosity fluid [27] to allow the device to reach a high value of sensitivity (2.5 kPa^−1^) and deliver a rapid response (of the order of tens of milliseconds) following mechanical stimuli. The high sensitivity of the sensor gives a complete signal of the waveform [28], even when the user reaches only the partial flattening of the artery. It does so by applying minimal pressure to it, which is crucial for the accurate calculation of hemodynamic parameters, especially when the heart rate is increased during exercise or in the case of mild tachycardia [29]. This feature, in combination with the high sensitivity of the device, facilitates patient comfort during continuous long-term monitoring.

## 2. Sensing Principle

**Figure 1:**
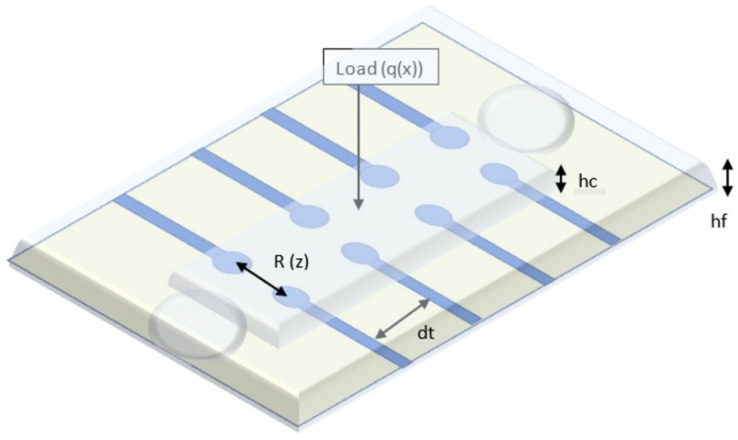
Schematic view of the blood pressure sensor.

Our sensor embeds a microfluidic layer located between two sensitive, transparent membranes (PDMS, PET). The microfluidic layer determines impedance changes from the deformation of the membrane when external pressure is applied [30]. The relationship between the applied pressure and the resistance of the device can be theoretically modeled by analyzing the deformation of the membrane under external pressure and the corresponding microfluidic resistive variation [31,32]. Each transducer of the device can be treated exclusively as a resistor, as shown in Figure 1. In order to eliminate interference from the working environment, a resistive detection was chosen instead of a capacitive one [33]. The resistance of a transducer is related to the microchannel height.

(1) $$R\approx \frac{\rho_{E}\cdot W_{m}}{d_{t}\cdot h_{f}\left(z\right)}$$

where *ρ_E_* is the electrical resistivity of electrolyte; *W_m_* is the microchannel width; *d_t_* is the transducer spacing (between two vicinity transducers); and *h_f_* is the microchannel height, which is a function of the deflection *z* of the microstructure at the location of the transducer. Based on our simulations, taking into account the conductivity of the electrolyte used (4 mS/cm), we can estimate that a distance between 2 neighboring transducers at least equal to the length of one transducer, is quite reliable for an accurate measurement.

## 3. Materials and Methods

The concept of the proposed flexible pressure sensor is as follows: between 2 polymer layers a microfluidic channel is aligned on configurated metal transducers (Au). The microfluidic channel is filled with an electrolyte so that volume changes from the application of external forces (waveform of the human pulse) in the channel are felt by the transducers. The substrate on which the metal transducers are configured is PET. The second layer consists of PDMS. It contains the microfluidic channel to be filled with electrolyte. The Au transducers are configured to form pairs of resistors. The location of the sensor determined the choice of layout with four pairs of transducers. This option was chosen so that when the sensor was placed on the radial artery of the wrist, it could capture the blood pressure waveform better. A sensor with a single pair of transducers would have made the measurement less precise, due to the lack of accuracy in positioning the sensor on the radial artery.

**Figure 2:**
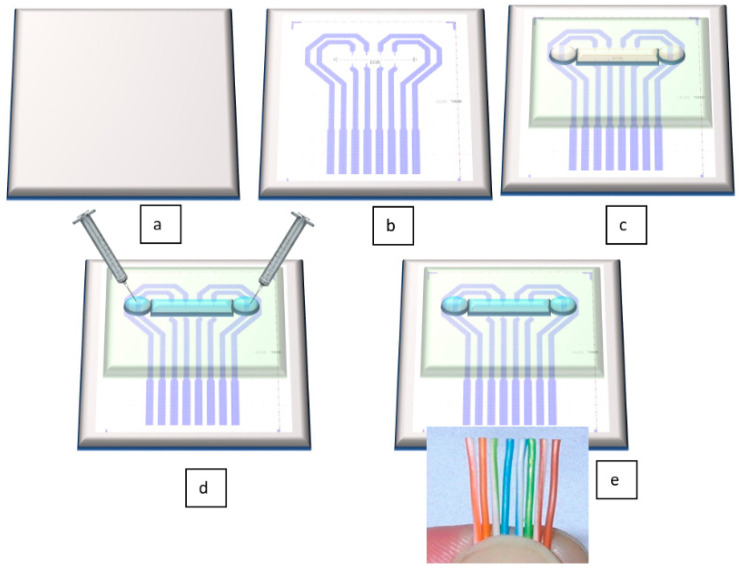
Schematic view of the technological flow for flexible pressure sensor fabrication: (**a**) PET foil is prepared for metal deposition, (**b**) Au layer is configured and deposited by DC sputtering; (**c**) PDMS membrane is formed together with microfluidic channel and attached through APTES (3-aminopropyltrimethoxysilane)/PDMS and GPTES (3-Glycidoxypropyltriethoxysilane) methods onto the PET foil [34]; (**d**) the channel is filled through its reservoirs with electrolyte; (**e**) electrical connections are made by wire bonding.

The novelty of our proposed sensor consists in its unique technological flow. In contrast to the results mentioned in the above citations, this flow has the undeniable advantages of using cheap, flexible, biocompatible materials, and having significantly lower costs than existing methods. Figure 2 depicts very well the technological flow for our sensor. All fabrication steps are described in detail in Section 3.2. Fabrication Technology.

### 3.1. Simulation

Since the preliminary tests showed encouraging results, a Finite Element Method (FEM) simulation model was deployed to test the sensor’s working principle and to optimize its outputs, especially in terms of sensitivity. The main aim of the modelling approach was to increase the variation of the resistance between a pair of electrodes, representing the sensor’s sensitivity, for low values of the channel’s deformation from the applied pressure. The chosen simulation tool was COMSOL Multiphysics^®^ software package, through its Electric Currents interface. This module computes the electric field, electric currents and the electric potential distribution within the analyzed model. The initial design of the electrodes and fluidic channel was considered for the virtual model development. The required materials and their relevant properties were defined for each of the elements that make up the pressure sensor. The material properties of the electrolyte [35] were those measured in the experiments (conductivity = 500 μS/cm). The electrodes were defined as Au with a conductivity of 45.6 × 10^6^ S/m. To simplify the simulation model, a single pair of electrodes forming a resistor was taken into account.

The simulation model considered the channel’s deformation from the pressure applied on top of it, while the electrodes were placed on the bottom part.

To take into account the effect of channel deformation on electrical conduction through the electrolyte and calculate the electrical resistance measured in this case, an imposed deformation on the channel was implemented. The deformation was defined transversely along the length of the channel to simulate the effect of the pressure/deformation by the blood vessel as accurately as possible. The degree of fluid-channel deformation was implemented progressively from 0 μm (un-deformed channel) up to 500 μm, relative to the initial height of the channel (800 μm) to capture and analyze different variations in blood pressure and its effect on channel deformation. In the initial sensor model evaluation, it was determined that four elements were the main parameters that could have important influences on the sensor’s outputs: the electrode’s width, the distance between the electrode’s terminals, the electrolyte’s electrical conductivity and the channel’s height. Therefore, the analyses focused on these four different aspects of the model to determine the influence of each on the efficiency of the pressure determinations: the width of the electrodes that make up the resistor (varying from an initial value of 100 μm, up to 1000 μm, in 6 steps); the distance between the ends of the electrodes that form a resistive pair (we considered the initial version, with the electrodes terminals placed at 1000 μm, a version with the terminals placed at 500 μm and a third version with the terminals placed at 1400 μm); and the variation of the electrolyte conductivity (basic version, 500 μS/cm; version 2, 11 mS/cm; version 3, 4 mS/cm). The basic model had the following parameters: electrode width, 100 μm; distance between terminals, 1000 μm; and electrolyte conductivity, 500 μS/cm. To capture clearly the effect of the four variations described above, each of the four parameters was modified while keeping the others at the basic values.

All analyses considered stationary studies and solvers, each of which had the same parametric sweep in 10 steps relative to the channel’s deformation.

**Figure 3:**
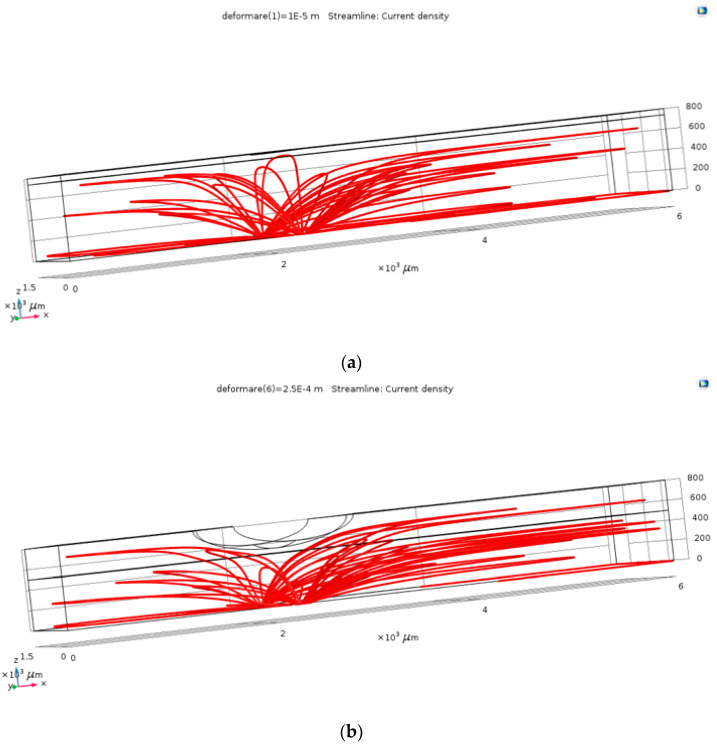
The current density within the channel, for the un-deformed (**a**) and deformed (**b**) channels.

The first step in the results processing consisted in visual plots of the current density through the electrolyte in the case of the un-deformed fluid channel and in one of the cases with deformation, in order to verify the operating principle of the pressure sensor. As resulted from Figure 3, the field lines of the current density (and of the electric field) follow the deformed geometry, so that, locally, at the level of the deformation, the conduction through the electrolyte decreases. This visual result proved that the simulation model correctly follows the phenomena, as it was estimated: channel’s deformation due to the blood vessel’s action increases the value of the electrical resistance between the two terminals.

Next, each of the simulation models included the numerical extraction of the relevant data. By means of a Global Evaluation interface, we extracted the resistance values between electrode terminals in each of the analyzed cases. Subsequently, we calculated the percentage variation of this resistance compared to the reference value of each case: the resistance of the un-deformed channel. Data from this parameter represented the most relevant information on the sensor’s sensitivity.

The simulations provided us with very important information regarding the sensor’s functionality with relevant directions toward its optimization. First of all, the model correctly captured the sensor’s principle of operation and showed good conformity with the preliminary tests performed on the initial design. Second, and most importantly, the simulations provided us with valuable suggestions for the proper way to optimize the sensor and increase its sensitivity.

From the point of view of the width of the metal electrodes, there was a significant increase in sensitivity with increasing electrode width. Optimum sensitivity occurred when the electrode width was in the 1000–2000 um range. The distance between the terminals introduced the expected variation of resistance, but the increase in sensitivity was relatively low: from 10% at the initial design) up to 14% when the terminals were 500 μm apart. The electrical conductivity of the electrolyte introduced significant variations in the determined resistance, but did not bring significant sensitivity benefits (from 10 to 12%). Therefore, the electrolyte type is not an important factor for sensor sensitivity, but it could be important if the range of the resistance needed to be modified. The height of the channel significantly affected the sensitivity of the sensor. As the channel’s height got lower, the sensor’s sensitivity increased because the proportion of the deformed volume in the fluidic channel was also larger. The results of the most important simulation cases are shown in Table 1, below.

Considering the conclusions detailed above, the final design of the sensor can include optimized parameters from each of the four analyzed cases. Such an approach can lead to the sensor’s sensitivity maximization. The optimal values of each parameter are

- Width of metal electrodes: 500–1000 μm;
- The distance between the terminals ends: 500 μm;
- Electrolyte with conductivity in the range of mS/cm; and
- Low-height microfluidic channel: 500 µm.

The information resulting from the simulations was used for the development of optimized pressure sensors for blood pressure read-out.

### 3.2. Fabrication Technology

The following technological flow describes in detail the materials and methods used to fabricate our flexible pressure sensor.

#### 3.2.1. Substrate Preparation and Au Deposition

**Figure 4:**
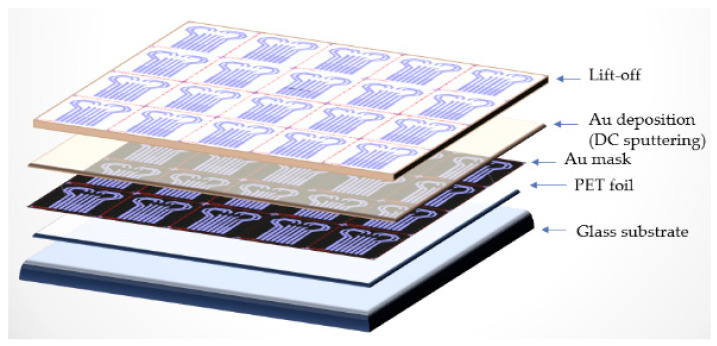
Technological flow for Ti/Au thin film deposition.

The substrate used was a 0.2 mm thick commercial PET (Polyethylene terephthalate) sheet. It was placed over a glass wafer as support for a lithographic process. O_2_ plasma dry cleaning was carried out using RIE (Reactive Ion Etcher) equipment. The process parameters were as follows: Power = 100 W; Pressure = 20 Pa; Oxygen flow = 40 cm^3^/min (sccm, standard cubic centimeters per minute); Time = 40 s. Standard lithographic technologies on 4 in. wafers were used, and the glass substrate helped to fix the PET foil, managing to finally obtain on a single glass wafer a number of 20 functional sensors. Ti/Au film (15/200 nm) was deposited by sputtering using Edwards dedicated equipment. The temperature of the substrate was usually below 100 °C during deposition. The Ti/Au deposition was performed in a pure argon atmosphere at a pressure of 5 × 10^−5^ to 2 × 10^−3^ torr, the power being 150 W (Figure 4). PET substrate was previously degreased with ultrasound in a diluted detergent solution of Sodium dodecylsulphate (SDS, 3% in 1:4 with H_2_O), rinsed in deionized water, then dried in a N_2_ atmosphere before being introduced into the storage room.

#### 3.2.2. Microfluidic Channel Formation and PDMS Membrane Fabrication

The material used for the microfluidic channel is SU-8 2050. The technological flux of the photolithographic process for SU-8 deposition on a glass substrate (10 cm × 10 cm) is as follows: Plasma O_2_ treatment for 5 min at 150 W, then deposition of SU-8 by centrifugation at 2000 rpm. Thermal treatment was followed at 65 °C for 5 min, then at 95 °C for 10 min, then exposure to UV M1 for 30 s and post-exposure treatment at 65 °C for 2 min and at 95 °C for 8 min. Then followed the development of the mR–Dev 600 exposure for 7 min and a thermal treatment at 150 °C for 20 min.

We obtained the PDMS membrane by pouring a mixture of 10 mL silicone elastomer +1 mL curing agent from Silgard over the mold of the microfluidic channel, then removing any O_2_ gaps by mechanical stirring, followed by a thermal treatment at 60 °C for 3 h.

#### 3.2.3. Bonding the Two Polymeric Layers and Filling the Microchannel

For bonding and sealing the PDMS membrane on PET/Au film, functionalization was applied by two methods: APTES ((3-aminopropyltrimethoxysilane)/PDMS) and GPTES (3-Glycidoxypropyltriethoxysilane). APTES: PDMS membrane functionalization done by rinsing with Et–OH and drying at room temperature followed by O_2_ plasma exposure with the following process parameters: Power, 20 W; Pressure, 20 Pa; and O_2_, 20 ccm flow. Functionalization was carried out with 5% APTES solution in C_2_H_5_OH for 20 min followed by O_2_ plasma exposure of PET film. Process parameters: Power, 20 W; Pressure, 20 Pa; O_2_, 20 sccm flow. GPTES: PET sheet functionalization done by rinsing in Et–OH and drying at room temperature and then for 5 min at 500 °C followed by O_2_ plasma exposure of PET foil.

**Figure 5:**
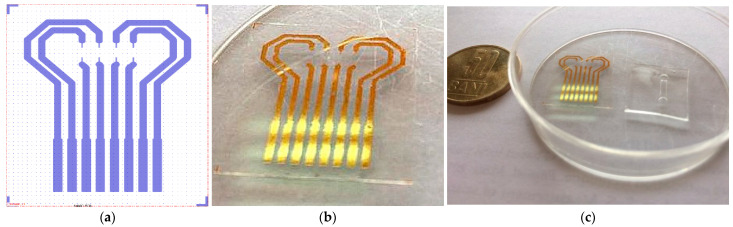
Au transducer layout (**a**)—fabricated Au transducer (**b**); Au transducer and microfluidic membrane before assembling (**c**).

**Figure 6:**
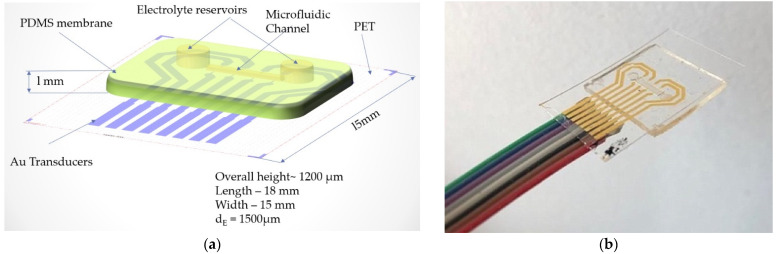
(**a**) Layout of the proposed flexible pressure sensor and (**b**) final sensor after assembly and wire bonding.

Process parameters: Power, 20 W; Pressure, 20 Pa; O_2_, 20 sccm flow. Functionalization of PET film was carried out with a 5% GPTES solution in C_2_H_5_OH for 3 h. Permanent contact of the flexible surfaces was achieved by overlapping the PDMS membrane with the PET film and left overnight at room temperature. In Figure 5, one can see the layout of the Au transducer layout (a) in comparison with fabricated Au transducer (b). Figure 6 shows the proposed layout and the final fabricated sensor.

**Figure 7:**
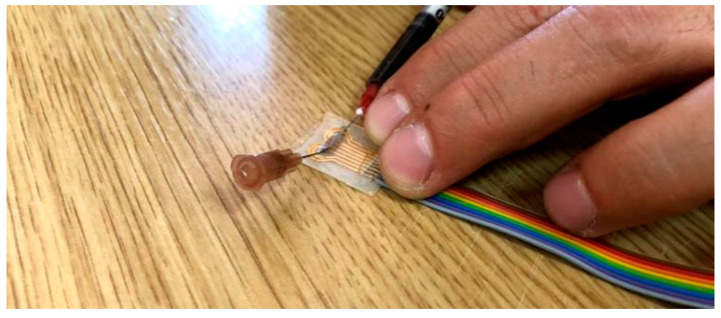
The process of filling the microfluidic channel with electrolyte solution through one reservoir while the air is being released from the second reservoir by a syringe needle.

The microchannel was filled with the electrolyte solution presented in Table 2 by punching a hole into one reservoir and injecting the fluid, while through the other hole air was removed by inserting a syringe. Once the channel was filled with the electrolyte, the two holes were plugged with 1 drop of PDMS, and then cured at 60 °C for 2 h (Figure 7).

#### 3.2.4. Preparation Instructions for S1

1. 4% PSS + 0.006% SWCNT + 0.266 ANI and 1% EG were placed in an Erlenmeyer flask and 17.67 mL H2O was added.
2. The solution was heated to 60 °C and alternatively added to the ultrasonic bath until the complete dissolution of the carbon particles. This can take up to 24 h.
3. The conductivity of the solution was 4 mS/cm at room temperature.

## 4. Results: Electrical Characterization for the Fabricated Flexible Pressure Sensor

The next stage in the optimization and characterization of the flexible pressure sensor was characterization by electrical measurements, which were validated in the laboratory. The electrical characterization was performed in several stages, as follows:

### 4.1. Direct Current Measurements (DC)

#### 4.1.1. DC-1. Flexible Pressure Sensor (FPS) Placed on the Artificial Arm 2 (IMT-Arm) with Pressure Applied from an OB1 Pulse Generator from Elveflow

A program was created in LabView to allow periodic DC resistance measurements to be performed for one of the sensor channels. To measure the sensor, a direct current of 10 µA or 1 µA (corresponding to a range of resistors measured of the order of MOhm, respectively tens of MOhm) was injected and the value of the voltage drop across the terminals was read, the resistance value being the ratio between this voltage and the injected current. The LabView program allows the acquisition of periodic measurements performed by the NI PXI-4071 multimeter module at the terminals where the sensor output is connected.

**Figure 8:**
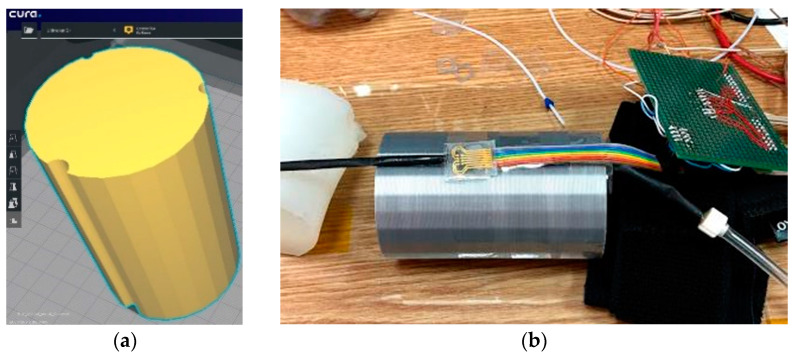
(**a**) Artificial arm layout (60 mm × 60 mm × 100 mm) and (**b**) Evaluation of the pressure Scheme 1. A flexible tube was placed on one of the channels of the artificial arm 2, which had thin walls through which the blood flow is simulated. Above it is the sensor connected to the measuring instruments.

For this version of the artificial arm, a 3D Printer (Ultimaker2+) was used. It is provided with 3 grooves of different diameters to accommodate flexible tubes through which blood pressure can be simulated. Figure 8 shows the 3D model. The sensor was attached to the surface of the artificial arm, over which the sleeve of a commercial sphygmomanometer was placed to induce measurable pressure values.

**Figure 9:**
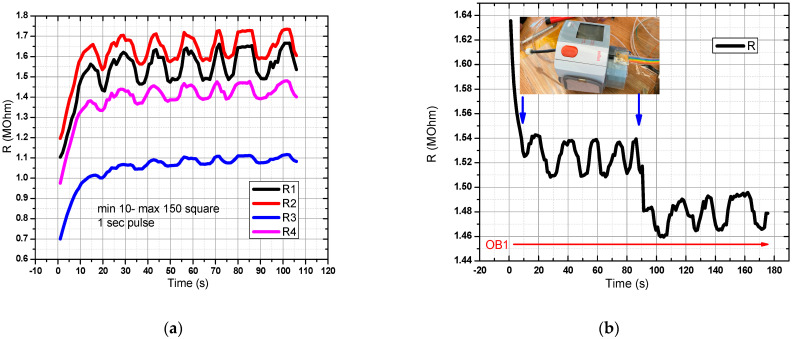
Evaluation of the pressure sensor characteristic in the final version (DC-1). In OB1 a rectangular pressure signal with a pulse width of 1 s was generated. The minimum pressure was 10 mm Hg and the maximum was 150 mm Hg. There is a good response from the sensor to the mentioned pressure values (**a**,**b**) Resistance variation for the pressure sensor manufactured in the DC-1 setup.

For the sensor in the graphical representation of Figure 9a in OB1, a flow rate profile was created, and a rectangular pressure signal with a pulse width of 1 s was generated. Acquisition time: 120 s. All 4 pairs of resistors were measured, and the response recorded in the range of resistance values of 1–2 MOhm. In Figure 9b, the digital sphygmomanometer was started at T = 0, and stopped at T = 90 s. The sensor was placed next to the sphygmomanometer for better accuracy in relation to the detected pressure changes. A decrease in the resistance value was observed when the digital sphygmomanometer was switched off. One can observe two decreases in the measured resistance value, the first arising from the pressure pulse drop coming from the flexible tube, and the second from the tension release from the digital sphygmomanometer. The first drop from the pulse in the flexible tube was greater than the drop arising from the sphygmomanometer release, thus showing that the sensor has a good sensitivity for measuring signals when prestressed from sphygmomanometer use. The slight drift can be attributed to the Joule effect and to slight ambient temperature changes.

#### 4.1.2. DC-2. (FPS) Placed on the Hand (Wrist Area)

**Figure 10:**
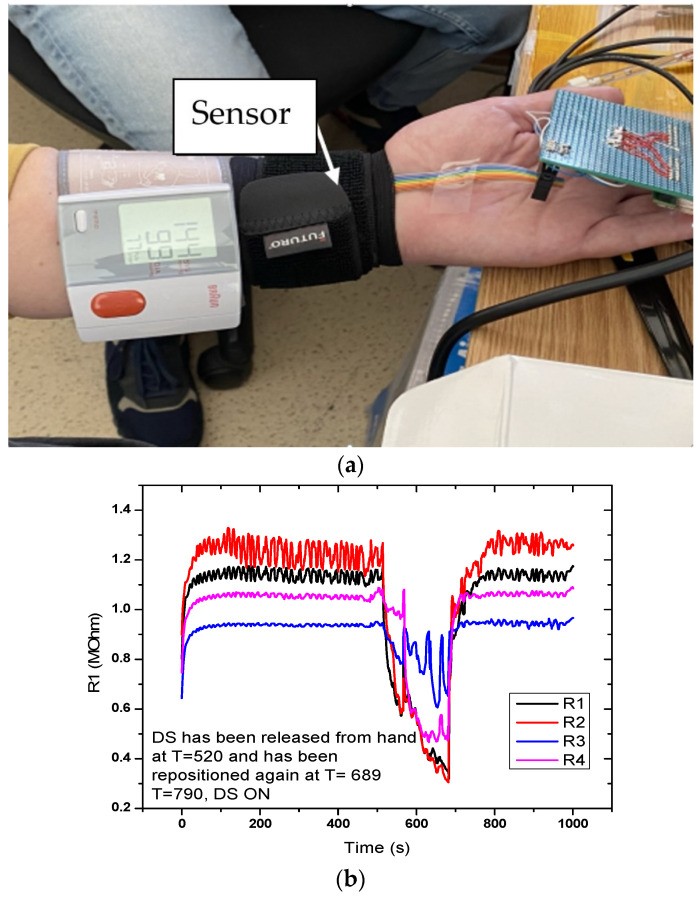
Measurement setup (DC-2) for evaluating the FPS. (**a**) The sensor is placed on a radial artery, covered by a Velcro band and immediately next to it is placed the digital sphygmomanometer; (**b**) Graphical representation of the measuring setup (DC-2) for the evaluation of the FPS.

Figure 10 shows the sensor response for the setup with the sensor placed on the hand. Measurement time was 1000 s. A sudden drop in the signal was observed at T = 520 s, when the digital sphygmomanometer was removed, but also when it was repositioned at T = 689 s. Graphical representation in the measurement setup (DC-2) for the evaluation of the flexible pressure sensor indicated that the sensor was sensitive to these variations. Measurements revealed that the sensor responded to pressure changes; however, the variations from the actual signal we wanted to monitor were small. Resistance values were in the MOhm range, which made it necessary to characterize the alternating current at different frequencies.

#### 4.1.3. DC-3 Direct Current Radial Artery Sensor (SAR)

For a better evaluation of the FPS, direct current measurements were performed by positioning the sensor on the human hand in the area of the radial artery. For better measurement and accuracy, the sensor was fixed with a sleeve on the artery. The purpose of this measurement was to capture the blood flow waveform.

**Figure 11:**
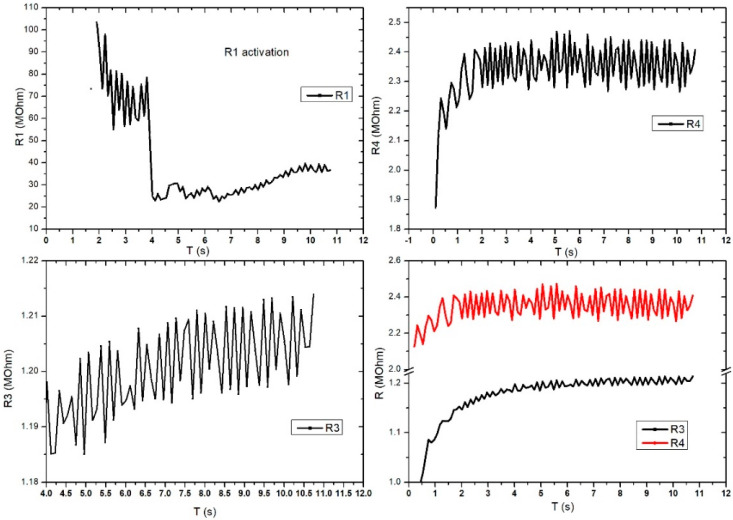
Graphical representation for the 4 pairs of resistors of the same sensor (sensor placed on the radial artery of the right hand-SAR). Acquisition time: 12 s.

**Figure 12:**
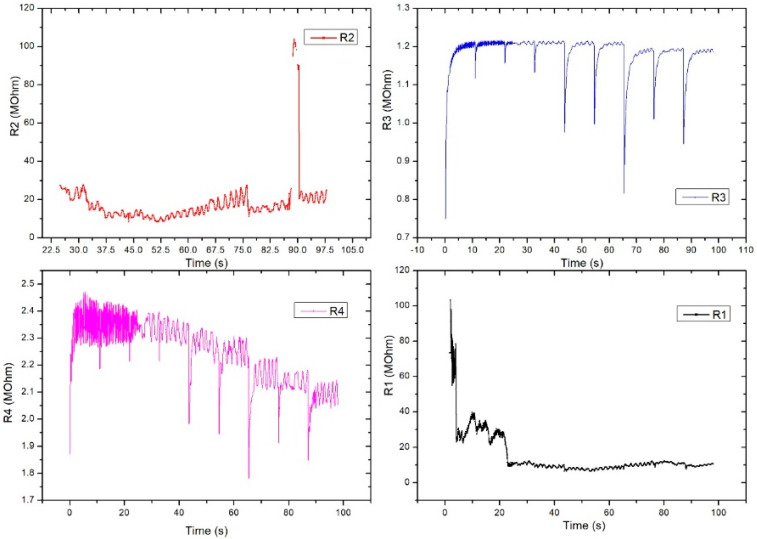
Graphical representation for the 4 pairs of resistors of the same sensor (sensor placed on the radial artery of the right hand-SAR). Acquisition time: 120 s.

Figure 11 shows the evaluation of the electrical characteristic for the FPS in radial artery sensor (SAR) operating mode. The measurements were performed in direct current, and the working methodology was similar to that used for DC2 except this setup gave up the digital sphygmomanometer. A periodicity of the obtained signal was observed at R3 and R4, which were similar to those of the heart rate-specific waveform. It is also interesting that R1 activates at values above 100 MOhm, followed by a stabilization trend in the range of 20–30 MOhm. Unlike Figure 11, Figure 12 presents the evaluation of electrical characteristics for the FPS in the working-mode sensor on the radial artery (SAR) in direct current, with a measurement and acquisition time of approximately 2 min. We wanted to show the stability of the sensor over a longer acquisition period. Following the continuation of R1 activation, as shown in the previous graph, we observed its stabilization in the range of 5–12 MOhm with a specific periodicity of the arterial blood pressure waveform.

R2 also showed a good signal and the inflections observed in the graph were taken at certain intervals (e.g., T = 25 s, T = 32.5 s, T = 75 s) and were caused by deliberate changes in the volunteer’s breathing or in controlled movements of the hand. Unlike R2, R3 had very good stability and periodicity around 1.2 MOhm. Periodic signal drops from 10 s to 10 s are due to acquisition stopping and resumption. R4 showed a clearer signal compared to the other pairs and showed large differences between the maximums and minimums (approximately 100 kOhm). The periodicity of the signal was also very good. The pairs of transducers behaved differently due to the positioning of the sensor on the artery area. This is the reason we used this specific sensor design: to make sure we got the waveform from at least one pair even if the sensor had a slight misplaced positioning. The highest value was considered.

### 4.2. Alternating Current Measurements (AC)

#### 4.2.1. AC-1 (FPS) Placed on MultiTest-i (MecMesin)

**Figure 13:**
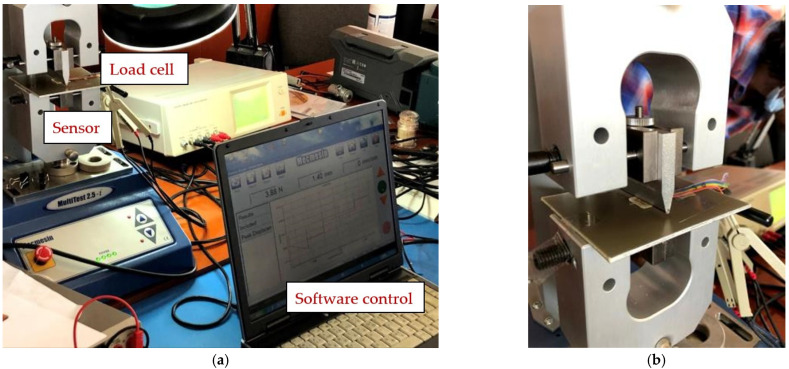
(**a**) Measurement setup (AC-1) for evaluating the FPS and (**b**) Measuring setup detail (AC-1).

**Figure 14:**
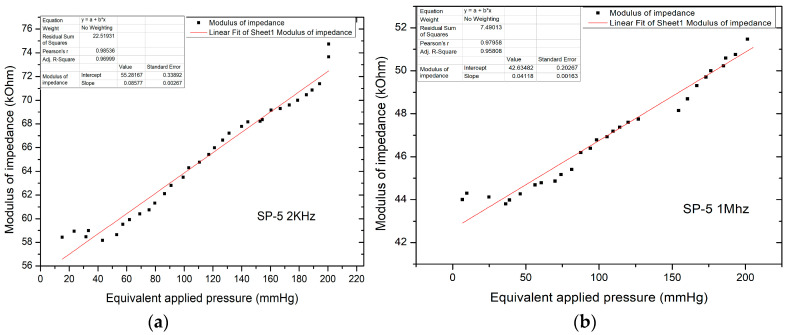
(**a**) Equivalent pressure applied vs. impedance modulus in AC-1 at 2 kHz; (**b**) Equivalent pressure applied vs. impedance modulus in AC-1 at 1 mHz.

The following presents the results of the electrical characteristics that validated the FPS fabricated by IMT through alternating current measurements. Figure 13 shows the measurement setup (AC-1) for evaluating the FPS. A MecMEsin MultiTest 2.5-i controlled force actuator (0.01–200 N) was used. The single-column MultiTest-i from MecMEsin Ltd. is a tensile and compression tester designed to apply tensile and compressive forces from a few mN to 5 kN. The sensor was placed in a mounting comprising a tank with a controlled actuation tip of the deformation at the sensor channel. The actuation was precisely controlled in the application of the equipment, and the electrical response resulting from the deformations in the channel was read through the RLC bridge. The signal parameters when reading the RLC are a 1 V amplitude sinusoidal signal, 2 kHz and 1 Mhz frequency. The acquired ones were represented by the module and the impedance phase read at the microfluidic channel of the FPS (Figure 14a).

Unlike direct current measurements, there was a significant decrease in the impedance modulus, now in the kOhm range, for pressure values up to 220 mm Hg, with impedance values in the modulus up to 75 kOhm. The characteristic shows the linear variation of the modulus of impedance with the applied pressure. The resulting sensitivity (characteristic slope) is 85.77 Ohm/mm Hg.

Figure 14b shows the equivalent applied pressure calculated from the value of the force applied for the SP-5 sensor at 1 MHz. Like the measurements performed at 2 kHz, here too there is a significant decrease in the impedance modulus which is now in the kOhm range, for pressures up to 200 mm Hg, with impedance values in the modulus up to 52 kOhm. The resulting sensitivity (characteristic slope) is 41.18 Ohm/mm Hg, which validated the sensor for the application.

#### 4.2.2. AC-2 (FPS) Placed on the Artificial Arm 2, Using the LRC Bridge and the OB1 Pulse Generator

**Figure 15:**
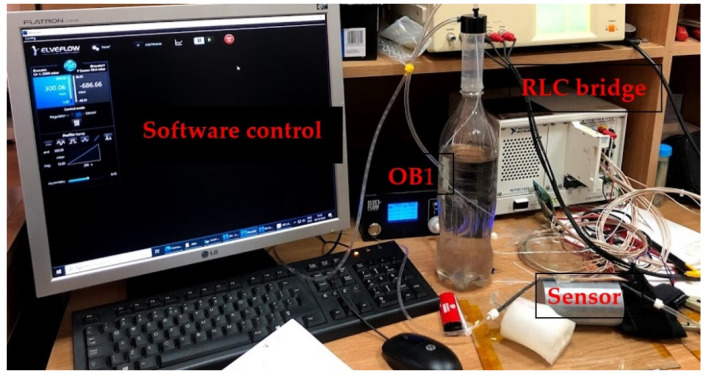
The AC-2 measurement setup used to validate the pressure sensor––final version.

Figure 15 shows the measurement setup for the AC-2 electrical characterization. The sensor was placed on the artificial artery of artificial arm 2 then connected to the RLC bridge for signal acquisition. The pulse generator was used to simulate blood flow through the artificial artery controlled through dedicated software. At OB1, a ramp signal was generated, starting from 0 to 300 mbar, the impedance modulus values being read at the RLC bridge. Measurements for the same sensor used in the AC-1 setup were performed at 2 kHz.

**Figure 16:**
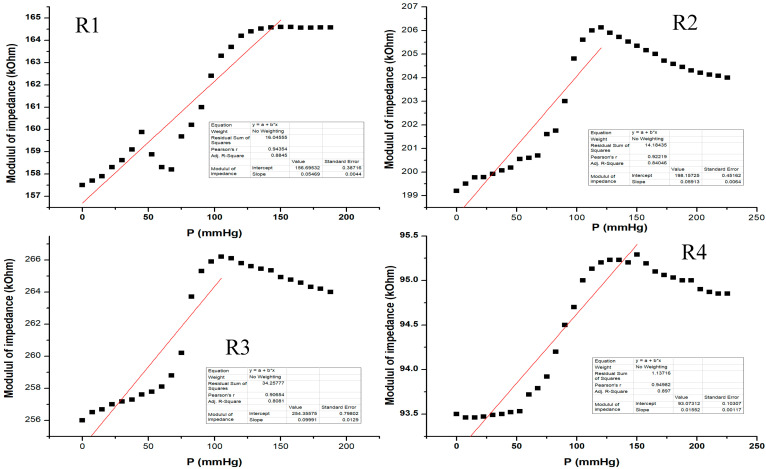
Equivalent pressure applied vs. impedance modulus in AC-2 at 2 KHz for all 4 pairs of transducers (R1–R4) from the same sensor.

Figure 16 shows the electrical characterization for our flexible sensor in AC-2 at 2 KHz. Data acquisition was performed for all 4 pairs of transducers from the same sensor. The sensor showed different response behavior depending on the pressure range. From the graphic, one can see that at low pressure values (from 0 to 50 mm Hg), the sensor behavior is not very steep and the slope is quite small. The sensor response becomes more linear at 50 to 120 mm Hg, and it seems that this would be the useful pressure range for the sensor to show the greatest response for all setups. Above 120 mm Hg, the sensor response no longer follows the previous characteristic because the applied pressure is great enough such that it no longer distributes its pressure toward the sensing element; thus, the sensor was no longer usable above 200 mm Hg in this measurement setup. The resulting sensitivity (characteristic slope) was around 57 Ohm/mm Hg for pressure values between 0 and 150 mm Hg.

## 5. Discussion

The technological flow for our fabricated pressure sensor represents a novelty that has undeniable advantages because it uses cheap, flexible, biocompatible materials and has significantly lower costs than existing methods. We consider that for fabrication, technological flow and electrolyte we obtained an optimized flexible pressure sensor to be placed on the wrist in the same bracelet as a read-out electronics module. This optimization consisted in simulation and technological processing, which was validated by electrical characterizations.

The deposited metal layer stood up very well inside our electrolyte media. The PDMS membrane proved to have very good mechanical properties intended for our application. Methods of functionalization of the substrates used by APTES (3-aminopropyltrimethoxysilane)/PDMS and GPTES (3-Glycidoxypropyltriethoxysilane)/PET were successfully used. The results of the functionalization of the two substrates demonstrated the adhesion of the PDMS to the PET film, thus achieving the proposed encapsulation process for our flexible pressure sensor.

To evaluate the sensor’s transfer characteristics (resistance versus pressure) and long-term functionality, a program was created in LabView to allow periodic measurements of DC resistance. For a better understanding of the physical phenomena behind the electrical characterization, different versions of the experimental setup were used. We used a pulse pressure generator and an artificial arm to mimic the measurement conditions for the acquisition of the wave function of human blood pressure as real as possible. We improved the setup by positioning the sensor on a human hand in the wrist area but also in the area of the brachial artery, thus managing to make measurements that allowed an electrical response as conclusive as possible for the final device. The Electrical characterization was performed in 5 different configurations using AC and DC measurements. The AC measurements for the fabricated pressure sensor resulted in impedance values at tens of hundreds of kOhm. The sensitivity of the sensor increased after the simulation and fabrication of the new modified sensors and obtained a value of 58 Ohm/mm Hg. We considered that the measurements in DC were also relevant for sensor evaluation and obtained a good sensitivity of it. However, AC measurements can lead to fabrication of fine resolution electronics, thus improving sensor sensitivity. AC results also allowed us to investigate more subtle changes in the electrical characteristics of the sensor pertaining to the nature of the electrolyte used in the microfluidic channel.

The measurements made on the fabricated sensor resulted in a linear increase in resistance to low pressure. High-pressure resistance characteristics were represented by a curve that increased exponentially with applied pressure.

Our sensor proved to have good sensitivity for pressure values between 0 and 150 mm Hg and was subject to repeated external forces, but further improvements related to detection algorithms for different pressure ranges and better electronics need to be done.

From preliminary observations, it appears that it would be necessary to correlate the sensor’s response with ambient temperature as it would seem from Figure 9, Figure 11, Figure 12, and Figure 16 that slight drifts from linearity might occur from slight changes in ambient temperature as measurements were not performed in a thermally controlled environment. In a future scenario, one might incorporate a temperature sensor to compensate for such effects.

The measured electrical values are very important for the selectivity of the sensor to be used in real conditions, for miniaturized electronic calibration, and for a wristwatch-type device.

Accessible and easy to use, our wearable pressure sensor could greatly increase access to hemodynamic investigations that could help diagnose and prevent heart disease. Moreover, this could contribute to the development of field studies and to the standardization of hemodynamic parameters to make studies in human patients much more feasible. The new sensor looks promising for further development and the new fluid-filled principle holds great potential, especially for beat-to-beat blood pressure measurement, but it will need further refinement before it can replace existing devices.

## Figures and Tables

**Table 1:** Studied parameters for channel deformation studies.

Channel’s Deformation (μm)	Initial Version		Electrodes Width 1000 μm		Electrolyte Conductivity 4 mS/cm		Channel Height 500 μm	
	Resistance (MOhm)	Sensitivity %	Resistance (MOhm)	Sensitivity %	Resistance (MOhm)	Sensitivity %	Resistance (MOhm)	Sensitivity %
0	2.9974	0.00%	0.89872	0.00%	0.37882	0.00%	3.1208	0.00%
50	2.9884	0.30%	0.89112	0.85%	0.37879	0.01%	3.1147	0.20%
100	2.9846	0.43%	0.90161	0.32%	0.37943	0.16%	3.1506	0.95%
150	3.0203	0.76%	0.89860	0.01%	0.3805	0.44%	3.2190	3.15%
200	3.0218	0.81%	0.91082	1.35%	0.38268	1.02%	3.3296	6.69%
250	3.0564	1.97%	0.93813	4.39%	0.38565	1.80%	3.4995	12.13%
300	3.0827	2.85%	0.94641	5.31%	0.38948	2.81%	3.7388	19.80%
350	3.1087	3.71%	0.97489	8.48%	0.39442	4.12%	4.1462	32.86%
400	3.1641	5.56%	1.01270	12.68%	0.40112	5.89%	4.8582	55.67%
500	3.3248	10.92%	1.12060	24.69%	0.42426	12.00%	N/A	N/A

**Table 2:** Electrolyte composition.

Solution 1 (S1)	Poly (Styrenesulfonate) (%)	SWCNT (Single Walled Carbon Nanotube) (%)	Aniline (%)	Ethylene Glycol EG(%)	Total H_2_O mL
Concentration (%)	4	0006	0.26	1	25 mL

## Data Availability

Not applicable.
